# Posterior Pituitary and Hypothalamic Neuronal Tumors in the 5th WHO Classification: Molecular Insights, Diagnostic Markers, and Clinical Management

**DOI:** 10.3390/ijms27136024

**Published:** 2026-07-04

**Authors:** Alexia Kesta, Omar Itani, Yahya Wehbeh, Dimitrios Kanakis

**Affiliations:** 1 Laboratory of Pathology, Department of Basic and Clinical Sciences, University of Nicosia Medical School, Nicosia 2408, Cyprus; kesta.a@live.unic.ac.cy (A.K.); itani.o1@live.unic.ac.cy (O.I.); wehbe.y@live.unic.ac.cy (Y.W.); 2 Centre of Neuroscience and Integrative Brain Research (CENIBRE), University of Nicosia Medical School, Nicosia 2408, Cyprus

**Keywords:** posterior pituitary tumors, hypothalamic neuronal tumors, pituitary neuroendocrine tumor, sellar/suprasellar masses

## Abstract

Posterior pituitary and hypothalamic neuronal tumors are uncommon sellar and suprasellar neoplasms that can mimic pituitary neuroendocrine tumors clinically and radiologically. The 5th edition World Health Organization classifications (Endocrine and Neuroendocrine Tumors) reinforce a lineage-based framework that separates anterior pituitary tumors from posterior pituitary and hypothalamic neuronal lineages, which is particularly important in hormone-negative lesions and limited tissue samples. This narrative review provides a practical, pathology-centered approach to classification by integrating key anatomic and radiologic clues with histomorphology and targeted immunohistochemistry. We highlight the value and limitations of thyroid transcription factor 1, outline a stepwise workflow incorporating anterior pituitary transcription factors and neuronal differentiation markers, and discuss when vasopressin immunostaining is informative. We also summarize selected molecular insights and clinical management considerations relevant to surgical planning and follow-up.

## 1. Introduction

Pituitary neuroendocrine tumors (PitNETs) arise from adenohypophyseal neuroendocrine cells and constitute the overwhelming majority of neoplastic sellar masses encountered in clinical practice [1,2]. Clinically, presentation ranges from local mass effects such as visual disturbance, headache, and hypopituitarism to hormone excess syndromes (e.g., Cushing syndrome), reflecting tumor lineage and functional status [2,3]. Yet the sellar and suprasellar compartment remains anatomically constrained and pathobiologically diverse, such that a meaningful subset of lesions are non-PitNET entities that can closely mimic PitNETs in symptoms and routine imaging [4,5,6].

This diagnostic convergence has practical consequences because MRI patterns overlap, tissue is often limited or fragmented (including frozen sections), and operative decisions often precede definitive histopathology [1,6,7]. Under these constraints, rarer lineages are vulnerable to default assignment as PitNETs or other common sellar masses when clinicoradiologic findings are equivocal, which can alter operative planning, expectations of endocrine behavior, and downstream surveillance strategies [1,8,9].

Against this backdrop, the WHO Classification of Endocrine and Neuroendocrine Tumors, 5th edition (WHO ENDO5) builds on the 4th edition and represents a pivotal refinement in pituitary tumor taxonomy, prioritizing lineage assignment using integrated histomorphology, transcription factor immunophenotype (PIT1, TPIT, SF1), and selected molecular features rather than relying on hormone stains alone [2,3,4]. This lineage-based framework has improved diagnostic precision and consistency across entities that were historically under-recognized, variably labeled, or inconsistently categorized in earlier schemes [2,3].

Among PitNET mimics, posterior pituitary tumors (PPTs) and hypothalamic neuronal tumors (HNTs) merit particular attention because they are uncommon in routine practice yet repeatedly generate diagnostic ambiguity [1,5]. Although arising from distinct lineages, these tumors frequently present with non-specific symptoms and overlapping imaging features, especially when tissue is limited and clinicoradiologic correlation is incomplete [1,5,8]. In WHO ENDO5, these lesions are grouped under ‘posterior pituitary and hypothalamic neoplasms’ and separated from anteriorly arising PitNETs. Within this framework, the pituicyte tumor family is supported by nuclear thyroid transcription factor 1 expression (TTF-1; NKX2-1 gene), enabling focused immunophenotyping across the posterior pituitary and infundibulum spectrum [3,5,10].

Existing reviews most commonly address posterior pituitary tumors or sellar neuronal tumors in isolation, leaving a practical gap for a unified WHO ENDO5-aligned, lineage-based approach to the major PitNET mimics in the posterior sellar and suprasellar region [3,5,9]. Accordingly, the aim of this narrative review is to provide a pragmatic WHO ENDO5-era approach for recognizing and classifying PPTs and HNTs by integrating anatomic context, clinicoradiologic patterns, endocrine phenotype, and a focused immunohistochemical workflow, with selective molecular testing in defined problem-solving settings to reduce misclassification and improve multidisciplinary decision-making [2,6,8].

## 2. Literature Retrieval and Source Selection

A literature search was performed to support this narrative review. PubMed/MEDLINE was searched from database inception through June 2026, supplemented by screening reference lists from relevant reviews, WHO classification materials, clinicopathologic series, and key primary studies. Core search terms included “posterior pituitary tumor,” “pituicytoma,” “spindle cell oncocytoma,” “granular cell tumor,” “ependymal pituicytoma,” “hypothalamic neuronal tumor,” “neurocytoma,” “gangliocytoma,” “PitNET,” and “TTF-1/NKX2-1,” combined with modifiers related to molecular findings, differential diagnosis, prognosis, and management. Priority was given to WHO 5th edition classification sources, contemporary clinicopathologic and molecular series, focused reviews, and diagnostically informative case reports or small series for rare entities. Articles were selected based on relevance to classification, histopathology, immunophenotype, molecular findings, differential diagnosis, clinical behavior, and management of posterior pituitary tumors and hypothalamic neuronal tumors. Because the aim was to provide a pathology-centered narrative synthesis rather than a systematic review or meta-analysis, formal risk-of-bias assessment and quantitative evidence synthesis were not performed.

## 3. Anatomical, Histogenetic and Classification Framework

### 3.1. Anatomy and Histogenetic Background

The pituitary gland is a compact endocrine organ at the skull base that regulates multiple physiologic systems through coordinated hormone secretion, and it is conventionally divided into the adenohypophysis, intermediate lobe, and neurohypophysis [4].

The neurohypophysis represents a downward extension of diencephalic neuroectoderm from the hypothalamic infundibulum and differs fundamentally from the adenohypophysis in both structure and function [4]. Rather than synthesizing hormones, the posterior pituitary serves as a storage and release site for hypothalamic neurohormones, most notably vasopressin and oxytocin, which are produced in hypothalamic nuclei and transported along axons to the pars nervosa for regulated systemic release [4,9].

The infundibulum forms the anatomic and functional conduit between hypothalamus and pituitary through tightly integrated neural and vascular pathways, a relationship that becomes clinically salient when lesions involve the stalk [4,6].

Arterial supply to the median eminence and upper infundibulum is largely provided by the superior hypophyseal arteries, supporting the portal and perivascular microenvironments that link hypothalamic signaling to pituitary regulation [4]. From a topographic standpoint, the posterior pituitary and infundibulum occupy the infrachiasmatic compartment beneath the optic chiasm, while more superior suprasellar relationships bring key arteries into close proximity with the optic apparatus and the floor of the third ventricle. This spatial organization is central to radiologic-pathologic correlation and helps explain symptom patterns related to chiasmal compression and hypothalamic or third ventricular involvement [4].

PPTs are thought to derive from pituicytes, the specialized glial cells of the neurohypophysis and infundibulum, and they characteristically show nuclear TTF-1 immunoreactivity when interpreted in the appropriate morphologic and anatomic context [1,5,10]. TTF-1‘s developmental association with ventral forebrain structures provides biologic support for its value as a lineage marker in the posterior pituitary and infundibulum spectrum [5].

WHO ENDO5 places strong emphasis on lineage biomarkers for classification. For adenohypophyseal PitNETs, the principal lineage-defining transcription factors assessed by immunohistochemistry are PIT1, TPIT, and SF1, which anchor tumor typing to cell lineage rather than hormone stains alone [3,11]. In contrast, PPTs typically lack adenohypophyseal lineage transcription factors and instead show consistent nuclear TTF-1 positivity, reinforcing their separation from PitNETs at the immunophenotypic level [3,9].

### 3.2. WHO 5th Edition; Pituitary Classification of Posterior Pituitary and Hypothalamic Neuronal Tumors

WHO ENDO5 expands and refines pituitary tumor taxonomy to improve recognition of rare entities, clarify lineage relationships, and support clinically meaningful categorization [3,11].

A defining shift is the prioritization of lineage and biomarker profiles integrated with morphology over hormone expression alone, which has practical implications for workup when imaging and symptoms are non-specific [3,11]. Within this framework, pituitary tumors are organized into anterior PitNETs, PPTs, HNTs, and other sellar region tumors including craniopharyngioma and pituitary blastoma [11].

WHO ENDO5 recognizes four core PPT types within the pituicyte tumor family, namely pituicytoma, granular cell tumor, spindle cell oncocytoma, and ependymal pituicytoma, which arise in the posterior pituitary and infundibulum and share nuclear TTF-1 positivity [5,9]. While nuclear TTF-1 strongly supports posterior pituitary/hypothalamic lineage in the appropriate setting, it is not absolutely specific to pituicyte-lineage tumors and should be interpreted with site and morphology, particularly on limited samples where TTF-1-positive ventral forebrain tumors (e.g., chordoid glioma) and metastatic pulmonary/thyroid carcinomas involving the sellar region remain important pitfalls [12,13,14].

HNTs are subdivided into neurocytoma and gangliocytoma and are typically centered in the suprasellar region, classically at or near the floor of the third ventricle. In selected settings, hypothalamic neuronal tumors may show TTF-1 expression and may exhibit immunoreactivity for hypothalamic hormones such as vasopressin, which can support recognition when integrated with site and morphology [15]. Operationally, WHO ENDO5 distinguishes PPTs and HNTs from PitNETs and other sellar lesions primarily through lineage-appropriate immunophenotype rather than anatomic location or hormone profile alone [5,11]. Here, we focus on the sellar/suprasellar neuronal tumors most often encountered in the differential diagnosis of PitNETs. WHO ENDO5-recognized posterior pituitary and hypothalamic neuronal entities and their defining lineage markers are summarized in Table 1.

## 4. Posterior Pituitary Tumors

### 4.1. Clinical and Radiologic Features

PPTs are rare, representing less than 1% of pituitary tumors in published estimates, and they most often present in adults with a peak incidence in the fifth to sixth decades [9]. Across aggregated cohorts, pituicytoma (PT) is frequently the most common subtype, comprising approximately half of reported PPTs, although subtype distribution varies across series and referral settings [16,17].

Clinical manifestations largely reflect local mass effect and stalk or optic pathway involvement, with common presentations including visual disturbance, headache, hypopituitarism, and mild hyperprolactinemia consistent with stalk effect [9,16,17]. Less commonly, adenohypophyseal hyperfunction syndromes have been reported in association with PPTs, including ACTH-dependent hypercortisolism and acromegaly, and these presentations should prompt careful assessment for coexisting sellar pathology or indirect endocrine mechanisms rather than assuming a “functioning” PPT [9,16]. Diabetes insipidus can occur but is uncommon at initial presentation in most series, despite the neurohypophyseal origin of these tumors [16,17].

High-resolution sellar magnetic resonance imaging (MRI) is the key investigation, and standard protocols typically include T1-weighted spin-echo sequences acquired in coronal and sagittal planes before and after gadolinium administration, complemented by coronal T2-weighted sequences [9,18]. PPTs lack distinctive radiologic findings, which contributes to frequent preoperative misclassification as nonfunctioning PitNETs (NF-PitNETs) and other sellar or suprasellar tumors [9,16,18]. Nevertheless, several suspicion-raising patterns have been described. PTs are often well-demarcated and may be centered in the sellar and or suprasellar region with intense enhancement, while granular cell tumor (GCT) can show characteristic T2 hypointensity and, in a subset, a “star-like crack sign” that may enhance after contrast administration [19,20]. For spindle cell oncocytoma (SCO), dynamic imaging may show hypervascular behavior with millimetric hypointense foci and linear signal-void areas on T1- and T2-weighted sequences [21]. These imaging features should be treated as hypothesis-generating rather than diagnostic, because validation is limited and overlap with common mimics remains substantial [9,20].

The overlap between PPTs and NF-PitNETs drives a high preoperative misdiagnosis rate, and although both entities are typically managed surgically, PPTs may pose distinct intraoperative challenges and may demonstrate different postoperative trajectories, including risks driven by vascularity, adherence, and the need to preserve the stalk and hypothalamic structures [16,17,22]. Radiomics and machine learning approaches have been explored as adjunct discriminators, with LASSO-based feature reduction and radiomic signatures showing potential to differentiate PPTs from NF-PitNETs and other common sellar lesions in retrospective and validation cohorts [23,24].

### 4.2. Histopathology and Immunophenotype

PPTs are generally regarded as low-grade neoplasms with indolent growth, although locally aggressive behavior and recurrent or persistent disease after surgery have been documented [11,25]. A practical unifying feature across PPT subtypes is pituicytic lineage with nuclear TTF-1 immunoreactivity in the appropriate anatomic and morphologic context [25,26,27]. Many PPTs are highly vascular, which is clinically relevant because vascularity and adherence can complicate resection and may contribute to subtotal excision in selected cases [25,28,29].

**Pituicytoma (PT):** PT is composed of spindle to bipolar cells arranged in short fascicles and or a storiform pattern, typically with low mitotic activity and low proliferative indices [28,30]. Across the pituicyte tumor family, morphologic overlap is common and reflects a spectrum of pituicyte differentiation rather than discrete endocrine lineages, which helps explain historical diagnostic variability among related entities [27,31,32]. Immunophenotypically, PTs show nuclear TTF-1 positivity with frequent S100 and vimentin expression, while GFAP staining is variable across series [28,32].

**Spindle cell oncocytoma (SCO):** SCO is characterized by spindle to epithelioid cells with abundant eosinophilic oncocytic cytoplasm that is enriched in mitochondria [27]. Typical immunophenotypic features include nuclear TTF-1 positivity with S100, EMA, and mitochondrial marker reactivity, and galectin-3 positivity is commonly reported, while GFAP is usually low or absent [27,28,31]. SCOs are frequently hypervascular and locally adherent, which can increase intraoperative bleeding risk and limit the likelihood of gross total resection in some cases [28,29].

**Granular cell tumor (GCT):** GCT shows polygonal cells with abundant granular eosinophilic cytoplasm and PAS-positive, diastase-resistant granules, and it often involves the stalk or posterior lobe [33]. Immunohistochemistry typically demonstrates S100 and CD68 positivity with supportive vimentin and α1-antitrypsin expression, consistent with the established pituicyte tumor family profile [33].

**Ependymal pituicytoma (EP):** EP is characterized by ependymal-type architecture including rosettes and perivascular pseudorosettes, and it retains the posterior pituitary lineage signature with nuclear TTF-1 positivity, while EMA positivity can be supportive in the appropriate morphologic setting [9,34]. This variant is exceptionally rare, with fewer than 20 cases reported in the recent literature [9]. Rare TTF-1-positive oncocytic sellar tumors with ependymal or follicular differentiation further illustrate the morphologic overlap within the pituicyte tumor family and the need for integrated classification [35].

From a surgical pathology standpoint, the main diagnostic challenge is separation of PPTs from more common sellar/suprasellar lesions on small specimens, particularly nonfunctioning PitNETs (including oncocytic variants), meningioma, schwannoma/other spindle-cell lesions, solitary fibrous tumor, and “sellar glioma” spectrum lesions (including pilocytic/pilomyxoid astrocytoma and, in the anterior third ventricle, chordoid glioma) [13,36,37]. Practical steps that reduce error include (i) prioritizing tissue triage for permanent sections and immunohistochemistry rather than relying on frozen morphology alone, (ii) using touch imprints/smears to preserve tissue when feasible, and (iii) applying a rapid, lineage-oriented approach on permanent sections (TTF-1 ± PitNET transcription factors ± targeted subtype markers) to secure an integrated diagnosis [38].

### 4.3. Molecular Pathogenesis

Molecular and epigenetic data increasingly support the concept that PPTs form a biologic spectrum with subtle methylation separation but meaningful differences in mutational profiles and copy-number burden. In the largest integrated series to date (47 tumors), genome-wide methylation differences across posterior pituitary tumor histologies were reported as subtle, consistent with shared lineage; however, targeted sequencing identified recurrent enrichment of MAPK/PI3K-pathway alterations within a pituicytoma/SCO-enriched molecular group (including FGFR1, HRAS, BRAF, NF1, and other pathway-related alterations), whereas many tumors in a granular-cell-enriched group lacked identifiable driver mutations by panel sequencing [31]. These findings complement prior reports of MAPK activation in SCO and pituicytoma (including HRAS and other MAPK-related events) and support the frequent observation of downstream pathway activation by immunohistochemistry (e.g., phosphorylated ERK) in subsets [28,31,39]. Additional clinicopathologic and genetic series have further supported subtype-associated differences within the posterior pituitary tumor family [40].

While molecular testing is not required for routine diagnosis in most cases, these data support reserving broader profiling for scenarios where risk stratification or additional treatment options are being considered (e.g., residual non-resectable disease, multiple recurrences, or unexpectedly aggressive clinical behavior), where identification of actionable MAPK/PI3K alterations could be clinically useful in selected patients [31,39].

### 4.4. Clinical Behavior and Prognosis

PPTs are typically indolent and are most often associated with favorable tumor-specific outcomes, but clinically meaningful recurrence or persistent disease is well documented, particularly with longer follow-up [25,41,42].

Recurrence or persistent disease has been reported in a substantial minority overall, and rates vary by subtype, with SCO repeatedly enriched among cases that recur or progress [31,43,44]. A key prognostic feature is that relapse can be delayed, with one large molecularly characterized cohort reporting a median time to recurrence for SCO of several years and a wide range extending beyond 9 years, supporting the need for prolonged surveillance even after an initially stable course [31,42]. In contemporary clinical cohorts, tumor-specific survival appears excellent, with a recent single-center series reporting 100% tumor-specific survival at 5 years, while overall survival is influenced primarily by non-tumor comorbidities [41].

Reliable histopathologic thresholds that consistently predict aggressive clinical behavior across PPT subtypes are not established, and commonly reported parameters such as proliferative indices have not shown robust separation of recurrent versus non-recurrent groups in pooled clinical datasets [28,29,43]. Emerging evidence suggests that molecular features may better stratify risk, with chromosomal copy number imbalances associated with shorter progression-free survival in posterior pituitary tumors, particularly within the combined pituicytoma and SCO spectrum [31]. In the same cohort, MAPK and PI3K pathway mutations were frequent in a subset but did not independently correlate with progression, indicating that pathway activation alone is not a sufficient prognostic surrogate in current datasets. These data support a model in which prognosis reflects a convergence of subtype biology and genomic stability, rather than histologic grade alone [21,31,39].

Morbidity is clinically important in PPTs because endocrine dysfunction and related metabolic sequelae may persist long term even when tumor control is achieved. In a recent cohort study, endocrine dysfunction largely persisted or worsened over follow-up and patients demonstrated progressive weight and BMI increases, most pronounced in those with granular cell tumors, underscoring hypothalamic-pituitary axis vulnerability in this anatomic compartment [41,42]. Similarly, broader series emphasize that long-term morbidity often reflects persistent hypopituitarism and water balance disturbance in a subset, rather than malignant transformation [25,28,42].

Overall, prognosis in PPTs is best framed as excellent tumor-specific survival with variable risk of delayed recurrence and substantial potential for chronic endocrine morbidity, which together justify sustained follow-up strategies that are tailored by subtype and emerging molecular risk signals.

### 4.5. Clinical Management

PPTs are managed primarily with surgical resection, and accurate subtype-level pathologic classification is clinically relevant because operative vascularity, adherence patterns, and recurrence tendencies influence both surgical planning and postoperative surveillance [25,37].

In practice, subtype-level expectations can be communicated preoperatively and used to structure surveillance. Tumors within the pituicytoma/SCO spectrum are often described as hypervascular and variably adherent to surrounding structures, which can reduce the feasibility of gross-total resection and thereby increase reliance on structured long-term imaging follow-up when residual disease is present [22,26,38]. Among PPTs, SCO has shown higher recurrence/progression rates in pooled analyses, particularly following subtotal resection, supporting a lower threshold for closer postoperative MRI surveillance in this subtype and in any case with residual tumor [43,44]. Emerging molecular evidence also suggests that chromosomal copy-number imbalances—reported more frequently in pituicytoma/SCO-enriched molecular groups—track with shorter progression-free survival and may justify intensified follow-up when identified [31].

In contrast to a typical PitNET, preoperative planning should anticipate the possibility of hypervascularity and firm adherence, particularly in PT and SCO, which can alter approach selection and hemostatic strategy [43,45]. When imaging suggests hypervascularity, including intense enhancement or intratumoral flow voids, case literature supports consideration of adjunct measures such as preoperative embolization in selected patients, recognizing that evidence remains limited to small series and reports [45,46].

For pituicytoma (PT), available retrospective evidence supports maximal safe resection as the primary disease-control strategy, with substantially lower recurrence after gross-total resection than after non-gross-total resection in aggregated patient-level analyses. Tumor size influences the feasibility of complete resection, and when gross-total resection is not achievable, adjuvant radiotherapy has been proposed for residual or progressive disease in selected scenarios, with the decision individualized to risk profile and anatomic constraints [25,45].

Reported radiation approaches for residual or recurrent PPTs include fractionated radiotherapy and stereotactic radiosurgery, typically selected according to residual volume and proximity to the optic apparatus, but the evidence base is retrospective and heterogeneous [45,47]. Accordingly, management is best individualized through multidisciplinary discussion among neurosurgery, radiation oncology, neuropathology, and endocrinology, with structured long-term radiologic follow-up.

For SCO, pooled analyses indicate a clinically challenging course in a substantial subset, with recurrence or progression frequently following subtotal resection, supporting a management strategy centered on maximal safe resection and consideration of adjuvant stereotactic radiosurgery for residual or recurrent disease. Long-term surveillance is particularly important in SCO because recurrence risk is not determined by low-grade histologic appearance alone and is tightly linked to operative constraints [43,44,47].

Management principles for other PPT subtypes remain less well defined because evidence is limited and largely case based, but symptom-driven intervention and individualized decisions around residual tumor and surveillance are common practice patterns. Across PPT subtypes, postoperative care should include endocrine and neuro-ophthalmologic monitoring, because endocrine dysfunction commonly persists or worsens after surgery and can contribute substantially to long-term morbidity [25,41]. The key histopathologic, immunophenotypic, molecular, behavioral, prognostic, and management features of WHO ENDO5-recognized posterior pituitary tumor subtypes are summarized comparatively in Table 2.

## 5. Hypothalamic Neuronal Tumors

### 5.1. Clinical and Radiologic Features

HNTs are exceptionally rare in the sellar and suprasellar region, and epidemiologic generalizations are constrained by the predominance of small series and case-based literature [48]. Within the best-characterized modern cohort of sellar neurocytoma, the dominant presenting clue is hyponatremia, reported in 73% of patients, emphasizing that electrolyte disturbance can be a more informative preoperative signal than imaging pattern alone [49]. In an additional clinicopathologic series of sellar/suprasellar neurocytoma, patients were typically young to middle-aged adults, often presenting with headache and visual symptoms, and preoperative imaging was commonly interpreted as pituitary adenoma with otherwise non-specific endocrine profiles [50].

Sellar gangliocytomas are likewise rare, but a focused review of “gangliocytomas of the sellar region” indicates that more than 150 cases have been reported, most frequently in association with an adenohypophyseal PitNET component [51]. This composite pattern has clear clinical implications, because the endocrine phenotype in many reported cases reflects the PitNET component, and acromegaly is repeatedly highlighted as the most frequent hyperfunction syndrome, with additional presentations including hyperprolactinemia and Cushing disease [51,52].

On MRI, both sellar neurocytoma and sellar gangliocytoma may appear as solid sellar or suprasellar masses that overlap with more common entities such as PitNET, germinoma, and other hypothalamic region lesions, contributing to frequent preoperative misclassification [49,50,53]. For sellar neurocytoma, a reproducible imaging tendency described in the largest contemporary cohort is growth toward the dorsal region, with a tendency for lateral invasion on preoperative imaging analysis [49]. For mixed gangliocytoma-PitNET, radiologic descriptions often include parasellar or cavernous sinus infiltration in compiled series, further increasing overlap with invasive PitNET patterns and underscoring the need for tissue-based classification [52].

### 5.2. Histopathology and Immunophenotype

HNTs of the sellar and suprasellar region appear to span a spectrum of neuronal differentiation, from neurocytoma to gangliocytoma. Notably, many reported gangliocytoma cases occur as mixed gangliocytoma–PitNET lesions, making this a recurrent diagnostic pattern [48,51,52].

**Sellar neurocytoma:** Sellar neurocytoma is a neuronal tumor composed of small, relatively uniform cells arranged in a sheet-like, monotonous architecture within a delicate fibrillary neuropil background. Immunophenotyping in sellar/suprasellar neurocytoma demonstrates a neuronal profile, including synaptophysin positivity, and in a clinicopathologic series additional markers reported as positive include calretinin, chromogranin A, and vasopressin, with focal staining for NeuN and TTF-1 in some cases [50]. Importantly, pituitary lineage transcription factors and anterior pituitary hormones are reported as negative in sellar/suprasellar neurocytoma, which is diagnostically useful when differentiating these tumors from PitNETs and from composite lesions. This interpretation is further supported by a series of hypothalamic vasopressin-producing tumors, in which sellar neurocytomas showed strong vasopressin immunoreactivity and nuclear TTF-1 positivity, while adenohypophyseal biomarkers remained negative, supporting hypothalamic neuronal differentiation rather than pituitary epithelial lineage [15].

**Gangliocytoma:** Sellar gangliocytoma is characterized by large, mature ganglion cells within neuropil, with prominent nucleoli and Nissl substance, and is typically highlighted by synaptophysin and neurofilament immunoreactivity. In the vasopressin-producing hypothalamic tumor spectrum, gangliocytoma represents the more differentiated end, and neuronal cells may show TTF-1 and vasopressin immunoreactivity, supporting hypothalamic neuronal differentiation in appropriate clinicopathologic settings [15,53]. Because gangliocytomas frequently coexist with PitNETs, immunohistochemistry should explicitly document both compartments, with neuronal markers defining the ganglion cell tumor and pituitary lineage transcription factors applied to classify the PitNET component [51,54].

### 5.3. Molecular Pathogenesis

For HNTs, molecular evidence is still emerging and remains secondary to morphology and immunophenotype in routine classification, but recent work provides concrete signals that may become diagnostically useful in representative contexts [49,50].

In sellar neurocytoma, transcriptomic analysis in the largest contemporary cohort identified SSTR2 expression and reported a novel LMCD1-AS1:GRM7-AS1 fusion event, with additional findings suggesting increased expression of hypothalamus-secreted hormones, supporting a hypothalamic differentiation signature at the molecular level. The same cohort proposed that somatostatin receptor ligand therapy could represent a potential therapeutic option in selected cases, although clinical evidence remains limited and should be considered hypothesis-generating [49].

In sellar/suprasellar neurocytoma, targeted molecular testing reported the absence of several canonical alterations seen in other CNS tumor lineages (for example IDH mutation and 1p/19q loss), reinforcing that the molecular differential diagnosis should be interpreted in a site-specific and lineage-specific manner [50]. Although data specific to sellar/suprasellar neurocytoma remain limited, broader central neurocytoma studies have reported altered H3K27me3 expression as a potential epigenetic mechanism, and the relevance of this finding to hypothalamic/sellar neurocytoma requires further validation [55].

For mixed gangliocytoma-PitNET lesions, molecular generalizations are difficult because the biology is inherently biphasic, and genomic interpretation should be compartment-specific when testing is performed [51,52].

### 5.4. Clinical Behavior and Prognosis

Clinical behavior in HNTs is strongly shaped by two recurring patterns: (i) composite lesions that include a gangliocytoma component with an adenohypophyseal PitNET component, and (ii) functional hypothalamic-lineage tumors associated with vasopressin biology and hyponatremia [52,56]. Composite gangliocytoma-PitNET lesions are repeatedly reported as single sellar masses with endocrine abnormalities, and the endocrine phenotype commonly reflects the PitNET component rather than intrinsic hormonal secretion by the neuronal compartment [51,52]. Accordingly, clinical course and follow-up requirements may be driven by both components, particularly when hormonal hypersecretion persists or recurs despite radiographic control of the mass lesion.

A clinically important functional subset includes hypothalamic-lineage neuronal tumors associated with vasopressin immunoreactivity, where SIADH and persistent hyponatremia may dominate presentation and perioperative morbidity [49,56]. Within contemporary sellar neurocytoma cohorts, hyponatremia is repeatedly highlighted as a common presenting clue, reinforcing that electrolyte disturbance can be a practical suspicion-raising feature when imaging is otherwise compatible with a routine PitNET [49].

Overall prognosis for sellar and suprasellar neurocytoma aligns with the broader extraventricular neurocytoma literature, where durable control is often achievable, but recurrence can occur, particularly when gross-total resection is not feasible [57,58]. In sellar/suprasellar neurocytoma series, relapse has been reported after subtotal resection, supporting long-term imaging surveillance even when early postoperative findings appear stable [48,54]. For mixed gangliocytoma-PitNET lesions, tumor control is generally favorable with surgery, but persistent endocrine hypersecretion may require secondary interventions and prolonged endocrine follow-up [51,52].

### 5.5. Clinical Management

For sellar and suprasellar neurocytoma, the cornerstone of treatment is maximal safe resection, while recognizing that subtotal resection is common in this anatomic compartment and may carry a higher risk of later progression [50,57]. When residual tumor remains or recurrence occurs, adjuvant radiotherapy is a commonly reported escalation strategy, and pooled extraventricular neurocytoma data support the frequent use of postoperative radiation as part of contemporary practice patterns [57,58]. Chemotherapy has no established routine role in sellar or suprasellar neurocytoma, and systemic therapy decisions beyond surgery and radiation remain individualized and case dependent [58].

For pituitary gangliocytomas and mixed gangliocytoma-PitNET lesions, management is primarily surgical, often via an endoscopic endonasal transsphenoidal approach when feasible, with endocrine management guided by the functional profile of the PitNET component [51,52]. Because endocrine morbidity can be a dominant determinant of long-term outcome in composite lesions and functional HNTs, postoperative care should incorporate structured endocrine monitoring and long-term imaging surveillance tailored to residual disease status and endocrine activity [52,57].

For vasopressin-associated hypothalamic and sellar neuronal tumors, management requires integrated tumor-directed therapy and active endocrine co-management of sodium and water balance, because SIADH can persist or recur in association with residual disease [56]. Diagnostic evaluation and treatment of hyponatremia should follow structured guideline frameworks, including assessment of serum and urine osmolality and urine sodium with stepwise fluid and pharmacologic management when indicated [59]. The principal diagnostic, molecular, clinical, prognostic, and management features of hypothalamic neuronal tumors and related composite lesions are summarized comparatively in Table 3.

## 6. Integrated Diagnosis and Differential Diagnosis

### 6.1. Radiologic-Pathologic Correlation in Sellar and Suprasellar Masses

A practical workflow begins with anatomic localization on MRI to determine whether a lesion is centered in the adenohypophysis, the neurohypophysis or infundibulum, or the suprasellar hypothalamic and third ventricular region [18,60]. In this setting, MRI is essential for defining lesion epicenter, relationship to the stalk and posterior lobe, hypothalamic or third ventricular involvement, optic pathway compression, cavernous sinus extension, and operative anatomy. However, imaging should be regarded primarily as a localization and surgical-planning tool rather than a definitive classifier, because PPTs, HNTs, PitNETs, and other sellar/suprasellar mimics frequently show overlapping radiologic manifestations.

PitNETs are typically centered in the adenohypophysis, and dynamic and contrast-enhanced sequences highlight that enhancement behavior can differ from normal pituitary tissue, while signal intensity varies with hemorrhage, infarction, cystic change, and subtype-specific features [60,61]. Therefore, MRI should be interpreted in parallel with endocrine phenotype and pretest probability due to differing imaging features across functional and non-functional PitNET subtypes.

PPTs often present as well-circumscribed sellar or sellar-suprasellar masses, and pituicytoma in particular may show uniform and strong enhancement with prominent flow voids around the lesion in some cases, reflecting hypervascularity [9,60]. Subtype-linked imaging patterns such as a “star-like crack sign” for granular cell tumor can be suspicion-raising but should not be treated as diagnostic because overlap with common sellar mimics remains substantial [9,20].

HNTs are typically suprasellar and may extend toward parasellar compartments, and sellar neurocytoma is frequently misinterpreted preoperatively as PitNET on routine imaging [49,50]. In the largest contemporary sellar neurocytoma cohort, a recurring imaging tendency is growth toward the dorsal region, which can serve as a suspicion-raising feature when paired with the clinical clue of hyponatremia [50].

Multidisciplinary correlation is particularly important in rare and diagnostically challenging lesions, where surgical approach, endocrine assessment, and pathology interpretation must converge to avoid misclassification and inappropriate downstream management [37]. A key differential within the posterior pituitary is metastatic disease, which may present as a dumbbell-shaped lesion and is often associated with central diabetes insipidus or arginine vasopressin deficiency, with imaging clues including sellar bone erosion without sellar enlargement [60].

### 6.2. Immunohistochemistry-Based Diagnostic Algorithm and Common Pitfalls

**Figure 1:**
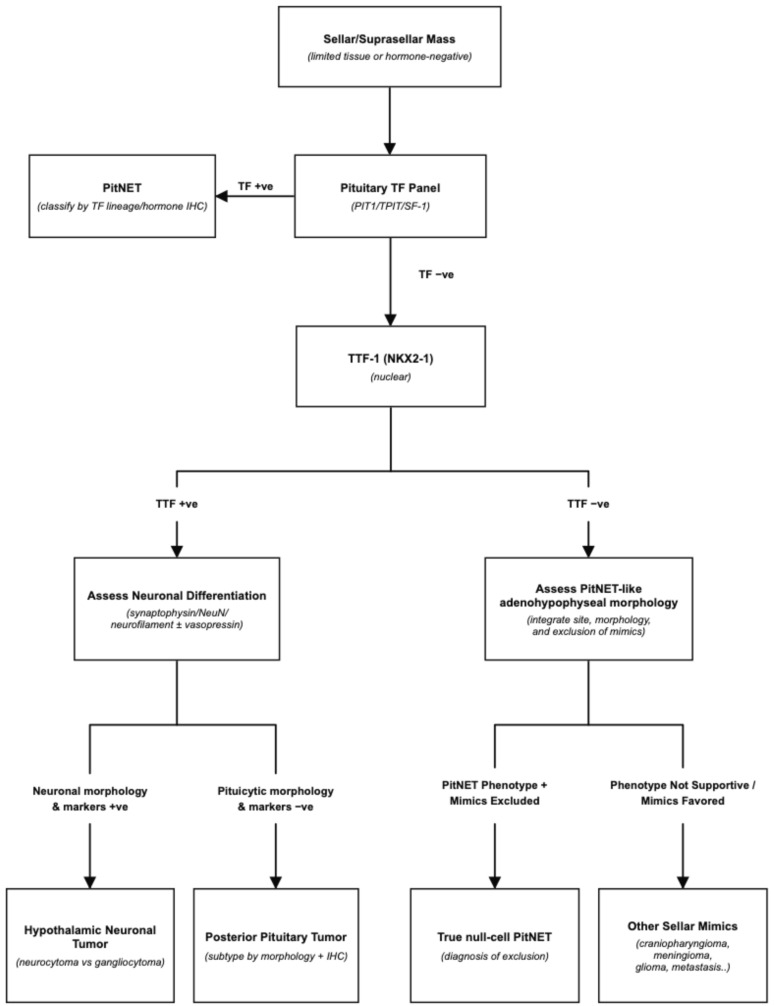
Lineage-based diagnostic workflow for sellar/suprasellar lesions with limited tissue or clinically absent hormone expression. Classification is guided by integrated assessment of morphology, anatomic/radiologic context, endocrine phenotype, pituitary transcription factors (PIT1/TPIT/SF1), TTF-1 (NKX2-1 protein), and neuronal differentiation markers (synaptophysin/NeuN/neurofilament ± vasopressin, when appropriate). Positivity for PIT1, TPIT, or SF1 supports PitNET lineage assignment, with hormone immunohistochemistry used for subtype confirmation when tissue permits. Conversely, a negative pituitary transcription factor and hormone profile should not be used alone to diagnose null-cell PitNET. In TF-negative lesions, nuclear TTF-1 positivity should prompt assessment for posterior pituitary tumor or hypothalamic neuronal tumor: pituicytic morphology with absent or limited neuronal differentiation supports posterior pituitary tumor, whereas neuronal morphology with supportive neuronal markers favors hypothalamic neuronal tumor. In TTF-1-negative lesions, true null-cell PitNET should be reserved for tumors with PitNET-like adenohypophyseal morphology after posterior pituitary tumors, hypothalamic neuronal tumors, and other sellar mimics have been excluded. TF: transcription factor; IHC: immunohistochemistry; TTF-1: thyroid transcription factor 1; PitNET: pituitary neuroendocrine tumor. Arrows indicate the sequential diagnostic workflow. Branch labels indicate positive (+ve) or negative (−ve) immunohistochemical or phenotypic findings.

Because PPTs and HNTs often lack specific MRI signatures, accurate classification under WHO ENDO5 depends on integrated interpretation of site, morphology, endocrine context, and lineage-based immunohistochemistry [11,27]. A pragmatic first-line panel for unusual sellar or suprasellar tumors may include PitNET transcription factors (PIT1, TPIT, SF1), TTF-1 (NKX2-1 protein), neuronal markers (synaptophysin, NeuN, neurofilament, with vasopressin when clinically relevant), and selected glial and epithelial markers (GFAP, S100, EMA) [12,27,37]. A practical workflow for hormone-negative or limited-tissue lesions is outlined in Figure 1.

Within this workflow, the TF-negative/hormone-negative branch should be interpreted as a differential diagnostic checkpoint rather than as an automatic endpoint for null-cell PitNET. True null-cell PitNET should be reserved for lesions that remain morphologically compatible with an adenohypophyseal PitNET after exclusion of TTF-1-positive pituicytic tumors, hypothalamic neuronal tumors, and other sellar mimics [3,11,12,27,35]. Conversely, nuclear TTF-1 positivity should prompt separation of pituicytic from hypothalamic neuronal differentiation using morphology and neuronal markers such as NeuN and/or neurofilament, with vasopressin reserved for selected hypothalamic-lineage contexts [15]. Synaptophysin alone is insufficient for this distinction because it is not specific for hypothalamic neuronal tumor. Therefore, in hormone-negative or TF-negative sellar/suprasellar tumors, the diagnosis of true null-cell PitNET should be made only after integrating morphology, site, endocrine context, and the targeted immunohistochemical panel rather than by immunonegativity alone.

Interpretation of immunohistochemistry integrated with histomorphology can be structured as follows:

- PitNET favored: PIT1 and or TPIT and or SF1 positivity supports adenohypophyseal lineage assignment, with hormone immunostains used for subtype confirmation and clinicopathologic correlation.
- PitNET TF negative: expand the differential to non-PitNET sellar entities and interpret TTF-1 and neuronal markers in the context of morphology and site:
  - HNT favored: strong and diffuse neuronal marker expression with architecture supporting neurocytoma or gangliocytoma, with subtype guided by neuronal cytology and organization.
  - PPT favored: nuclear TTF-1 positivity with absent PitNET transcription factors and absent or only limited neuronal marker expression, with subtype assignment relying on morphology and supportive markers such as GFAP, EMA, S100, and PAS.
- Recurrent or progressive PPTs: consider targeted molecular evaluation focused on MAPK and PI3K pathway-related alterations, including HRAS, BRAF, and FGFR1, as a problem-solving adjunct rather than a routine requirement.

## 7. Conclusions

PPTs and HNTs remain diagnostically important because they are uncommon and frequently present with non-specific clinical and imaging findings that overlap with PitNETs. WHO ENDO5 provides a practical framework that reduces this ambiguity by shifting classification toward an integrated, lineage-based diagnosis anchored in anatomic context, characteristic morphology, and transcription factor-based immunophenotyping rather than hormone stains alone. In routine practice, a minimal “lineage-first” approach—PitNET transcription factors (PIT1/TPIT/SF1), nuclear TTF-1, and neuronal differentiation markers (synaptophysin with NeuN and/or neurofilament, with vasopressin when clinically relevant)—offers the highest diagnostic yield for separating PitNETs from the PPTs and from HNTs, including composite gangliocytoma-PitNET lesions.

Clinically, management is driven by surgical feasibility and long-term endocrine morbidity as much as by histologic grade. For PPTs, hypervascularity and adherence can limit gross-total resection and contribute to delayed recurrence, particularly in spindle cell oncocytoma, supporting prolonged imaging surveillance and structured endocrine follow-up. Molecular data increasingly suggest that copy-number imbalance may stratify progression risk in subsets of PPTs, while MAPK/PI3K-pathway alterations may provide a rationale for selective profiling in recurrent/progressive or unresectable disease rather than routine testing in all cases.

Future progress will depend on multicenter clinicopathologic registries with standardized imaging, uniform immunopanels, and harmonized molecular profiling to validate radiologic suspicion features, refine risk stratification beyond proliferative indices, and define evidence-based indications for adjuvant radiotherapy and targeted or receptor-directed therapies in selected patients.

## Figures and Tables

**Table 1:** Posterior pituitary tumors and hypothalamic neuronal tumors in WHO ENDO5: diagnostic signatures. Abbreviations: TTF-1 = thyroid transcription factor 1; PitNET TFs = PIT1/TPIT/SF1; PAS-D = diastase-resistant PAS; NF = neurofilament.

Entity	Typical Epicenter	Hallmark Morphology	Core Lineage IHC	Key Rule-Outs (If Positive, Think)
**Pituicytoma**	Posterior lobe/infundibulum	Bland spindle cells; fascicular/storiform; hypervascular	TTF-1+, PitNET TFs−	- STAT6+ → SFT - PR/SSTR2A+ → meningioma - SOX10+ → schwannoma
**Spindle cell oncocytoma**	Posterior lobe/infundibulum	Spindle/epithelioid oncocytic cells	TTF-1+, PitNET TFs−	- PitNET TFs+ → oncocytic PitNET - PAX8 and/or CAIX+ → metastatic RCC
**Granular cell tumor (sellar region)**	Stalk/posterior lobe	Polygonal cells with coarse granules; PAS-D+	TTF-1+, PitNET TFs−	- Diffuse CD163/CD45-rich infiltrate → histiocytic/inflammatory process (interpret with clinical/radiology) - PitNET TFs+ → PitNET
**Ependymal pituicytoma (rare)**	Infundibulum/posterior region	Ependymal-like architecture (rosettes/pseudorosettes)	TTF-1+ reported, PitNET TFs−	Classic ependymoma context (site + broader profile) if true ependymoma markers/pattern dominate; avoid definitive call on crushed/limited tissue
**Sellar/suprasellar neurocytoma**	Sellar/suprasellar	Monomorphic neuronal cells with neuropil	Synaptophysin+, NeuN and/or NF+, PitNET TFs−	- PitNET TFs+ (PIT1/TPIT/SF1)+ → PitNET - OCT3/4 and/or PLAP+ → germinoma
**Gangliocytoma**	Hypothalamic-suprasellar ± sellar	Mature ganglion cells ± neuropil	Synaptophysin+, NF+	- PitNET TFs+ in a second component → composite gangliocytoma-PitNET - consider metastasis markers if atypical cytology
**Gangliocytoma-PitNET composite**	Sellar/suprasellar	Admixed neuronal + PitNET components	Neuronal markers + PitNET TFs define PitNET lineage	Document BOTH components and specify PitNET lineage by PIT1/TPIT/SF1 (not just “adenoma”)

Note: Arrows indicate the diagnostic implication of a positive marker or feature. The entity after the arrow should be considered or favored in the differential diagnosis; arrows are used as diagnostic shorthand and do not imply tumor progression, transformation, or causality.

**Table 2:** Comparative features of posterior pituitary tumor subtypes.

Entity	Hallmark Histology	Immunophenotype	Molecular/Pathway Features	Behavior and Management
**Pituicytoma**	Bland spindle/bipolar cells; fascicular or storiform growth; low proliferative activity	TTF-1+; S100+; vimentin+; GFAP variable	MAPK/PI3K-pathway alterations reported in pituicytoma/SCO-enriched molecular groups	Usually indolent; recurrence/persistence possible after incomplete resection; maximal safe resection and surveillance if residual/recurrent
**Spindle cell oncocytoma**	Spindle/epithelioid oncocytic cells; mitochondria-rich cytoplasm; often vascular/adherent	TTF-1+; S100+; EMA+; mitochondrial markers+; galectin-3 often+; GFAP low/absent	MAPK/PI3K alterations and copy-number imbalances reported in subsets	Higher recurrence/progression tendency, especially after subtotal resection; delayed relapse can occur; close MRI follow-up and selected radiosurgery/radiotherapy for residual/recurrent disease
**Granular cell tumor**	Polygonal cells with coarse granular eosinophilic cytoplasm; PAS-D+ granules; stalk/posterior lobe involvement	TTF-1+; S100+; CD68+; vimentin+; α1-antitrypsin+	Granular-cell-enriched tumors often lack identifiable panel-detected driver alterations	Usually indolent; symptom-directed surgery; endocrine and neuro-ophthalmologic follow-up, particularly for persistent pituitary-axis morbidity
**Ependymal pituicytoma**	Ependymal-like architecture; rosettes/pseudorosettes; very rare	TTF-1+; EMA may support diagnosis	Limited data because of rarity	Individualized management; long-term surveillance guided by residual disease, symptoms, and limited evidence base

Abbreviations: EMA, epithelial membrane antigen; GFAP, glial fibrillary acidic protein; MAPK, mitogen-activated protein kinase; PAS-D, diastase-resistant periodic acid-Schiff; PI3K, phosphoinositide 3-kinase; SCO, spindle cell oncocytoma; TTF-1, thyroid transcription factor 1.

**Table 3:** Comparative features of hypothalamic neuronal tumors and related composite lesions.

Entity	Hallmark Histology	Immunophenotype	Molecular/Pathway Features	Behavior and Management
**Sellar/suprasellar neurocytoma**	Uniform neuronal cells in sheets with delicate neuropil	Synaptophysin+; calretinin/CgA/vasopressin reported; NeuN and TTF-1 may be focal; PitNET TFs and anterior pituitary hormones−	SSTR2 expression and LMCD1-AS1:GRM7-AS1 fusion reported in the largest contemporary cohort; IDH mutation and 1p/19q loss absent in tested cases	Maximal safe resection; recurrence possible after subtotal resection; radiotherapy considered for residual/recurrent disease; somatostatin receptor ligand therapy remains hypothesis-generating
**Gangliocytoma**	Mature ganglion cells within neuropil; prominent nucleoli/Nissl substance	Synaptophysin+; neurofilament+; TTF-1 and vasopressin may be present in hypothalamic-lineage cases	Limited data; diagnosis remains primarily morphology/IHC-based	Usually favorable control with surgery; follow-up guided by residual disease and endocrine status
**Mixed gangliocytoma-PitNET**	Biphasic neuronal and adenohypophyseal components	Neuronal markers define gangliocytoma; PIT1/TPIT/SF1 classify PitNET component	Compartment-specific interpretation required because biology is biphasic	Surgery is primary; endocrine course often driven by PitNET component; prolonged endocrine follow-up may be needed
**Vasopressin-associated hypothalamic/sellar neuronal tumor**	Functional hypothalamic-lineage neuronal tumor; may overlap with neurocytoma or gangliocytoma morphology	Vasopressin+ in selected cases; TTF-1 may support hypothalamic lineage	Limited data; current interpretation remains clinicopathologic	SIADH/hyponatremia may dominate presentation; tumor-directed treatment plus active sodium/water-balance management

Abbreviations: CgA, chromogranin A; HNT, hypothalamic neuronal tumor; IDH, isocitrate dehydrogenase; IHC, immunohistochemistry; PitNET, pituitary neuroendocrine tumor; SIADH, syndrome of inappropriate antidiuretic hormone secretion; SSTR2, somatostatin receptor 2; TF, transcription factor; TTF-1, thyroid transcription factor 1.

## Data Availability

No new data were created or analyzed in this study. Data sharing is not applicable to this article.

## Abbreviations

The following abbreviations are used in this manuscript:

Abbreviation	Full term
ACTH	Adrenocorticotropic hormone
BRAF	B-Raf proto-oncogene, serine/threonine kinase
CD68	Cluster of differentiation 68
CgA	Chromogranin A
CNS	Central nervous system
CRH	Corticotropin-releasing hormone
EMA	Epithelial membrane antigen
EP	Ependymal pituicytoma
ERK	Extracellular signal-regulated kinase
FGFR1	Fibroblast growth factor receptor 1
GCT	Granular cell tumor
GFAP	Glial fibrillary acidic protein
GH	Growth hormone
HNT	Hypothalamic neuronal tumor
IHC	Immunohistochemistry
LASSO	Least absolute shrinkage and selection operator
MAPK	Mitogen-activated protein kinase
MRI	Magnetic resonance imaging
NF-PitNET	Non-functioning pituitary neuroendocrine tumor
NKX2-1	NK2 homeobox 1
PAS	Periodic acid-Schiff
PI3K	Phosphoinositide 3-kinase
PIT1	Pituitary-specific positive transcription factor 1 (POU1F1)
PitNET	Pituitary neuroendocrine tumor
PPT	Posterior pituitary tumor
PT	Pituicytoma
SCO	Spindle cell oncocytoma
SF1	Steroidogenic factor 1
SIADH	Syndrome of inappropriate antidiuretic hormone secretion
T1	T1-weighted magnetic resonance imaging
T2	T2-weighted magnetic resonance imaging
TF	Transcription Factor
TPIT	T-box pituitary transcription factor (TBX19)
TTF-1	Thyroid transcription factor 1
WHO	World Health Organization
WHO ENDO5	WHO Classification of Tumours: Endocrine and Neuroendocrine Tumours, 5th edition
